# Comparisons of Aconite with Apis and Gelsemium in Fever

**Published:** 1888-07

**Authors:** 


### COMPARISONS OF ACONITE WITH APIS AND GELSEMIUM IN FEVER.

Aconite.—Anguish, despair, restless tossing about during the fever; fears he will die; throws off the clothes; pulse full, hard, bounding; skin hot and dry. All ends in copious sweats.

Apis.—Fidgety, restlessness; wants to sleep, but so nervous cannot; or low, muttering delirium; Sopor. Chill begins in knees or abdomen about three p. m.; heat, with dry skin or occasional transient spells of sweating; desire to uncover; great oppression of the chest; skin hot in some places and cool in others. Pulse accelerated and strong; or debility shows itself; pulse wiry and frequent; imperceptible and intermittent. Apt to be thirstless.

Gelsemium.—Irritable, sensitive; children, sometimes wakeful; nervous, even threatened with convulsions; or drowsy, eyelids heavy, looks as if intoxicated; want to remain perfectly quiet (Bry.). Chill up and down back, followed by fever with increased drowsiness; pulse full, flowing. Sweat moderate, gradual, but giving relief.

